# Zinc in Wound Healing Modulation

**DOI:** 10.3390/nu10010016

**Published:** 2017-12-24

**Authors:** Pei-Hui Lin, Matthew Sermersheim, Haichang Li, Peter H. U. Lee, Steven M. Steinberg, Jianjie Ma

**Affiliations:** 1 Davis Heart and Lung Research Institute, The Ohio State University Wexner Medical Center, Columbus, OH 43210, USA; sermersheim.3@osu.edu (M.S.); Haichang.Li@osumc.edu (H.L.); Peter.Lee@osumc.edu (P.H.U.L.); 2 Department of Surgery, Division of Cardiac Surgery, The Ohio State University Wexner Medical Center, Columbus, OH 43210, USA; 3 Department of Surgery, Division of Trauma, Critical Care and Burn, The Ohio State University Wexner Medical Center, Columbus, OH 43210, USA; Steven.Steinberg@osumc.edu

**Keywords:** inflammation, immune response, anti-oxidant, tissue proliferation, matrix remodelling, TRIM family proteins, matrix metalloproteinase

## Abstract

Wound care is a major healthcare expenditure. Treatment of burns, surgical and trauma wounds, diabetic lower limb ulcers and skin wounds is a major medical challenge with current therapies largely focused on supportive care measures. Successful wound repair requires a series of tightly coordinated steps including coagulation, inflammation, angiogenesis, new tissue formation and extracellular matrix remodelling. Zinc is an essential trace element (micronutrient) which plays important roles in human physiology. Zinc is a cofactor for many metalloenzymes required for cell membrane repair, cell proliferation, growth and immune system function. The pathological effects of zinc deficiency include the occurrence of skin lesions, growth retardation, impaired immune function and compromised would healing. Here, we discuss investigations on the cellular and molecular mechanisms of zinc in modulating the wound healing process. Knowledge gained from this body of research will help to translate these findings into future clinical management of wound healing.

## 1. Introduction

Wound healing is a physiological response to injury that is essential across all tissue systems. Wound care is a major public health issue. Based on an economic evaluation of 2014 Medicare expenses, the total annual costs of all wound types, such as pressure ulcer, venous ulcers, diabetic foot ulcer, surgical and traumatic wounds and infection, ranged from $USA28.1 to $USA96.8 billion in the United States [1]. The burden of wound treatment remains a significant challenge due to an aging population and increases in diabetes and obesity, conditions that contribute to aberrant wound healing. The underlying complexities of the wound healing process include membrane repair, coagulation, control of inflammation, angiogenesis, cell proliferation, tissue remodelling and scar formation. These cumulative functions are integral for restoring tissue architecture.

Zinc is an essential micronutrient, present at less than 50 mg/kg, in the human body. It is important for human health and disease due to its critical roles in growth and development, bone metabolism, the central nervous system, immune function and wound healing [2]. Zinc is a vital cofactor for the function of more than 10% of proteins encoded by the human genome (~3000 proteins/enzymes) [3,4]. Zinc-dependent proteins play numerous indispensable roles within cells, such as transcriptional regulation, DNA repair, apoptosis [5,6], metabolic processing [7], extracellular matrix (ECM) regulation [8] and antioxidant defence [9].

Low concentration of zinc is found intracellularly across all tissues as a divalent cation incapable of passive diffusion across cell membranes. As such, the cellular homeostasis and bioavailability of zinc is tightly controlled through transcriptional regulation, ion transporters and compartmental stores [10,11,12,13]. Metabolically active, labile zinc is present inside cells at pico- to nanomolar concentrations with extracellular concentrations in the sub-micromolar to micromolar range [14]. The zinc transporter protein (ZIP) family zinc uptake/transporter proteins, particularly ZIP4, are responsible for zinc uptake from the extracellular milieu or intracellular vesicles [15]. Cytosolic free zinc ions have recently been identified as secondary messengers, similar to calcium ion transients, capable of interacting with target proteins in order to regulate many signal transduction pathways [16,17]. In this regard, zinc availability and regulation constitute an important component in cell physiology.

A common cause of zinc deficiency is malnutrition. Dietary zinc is absorbed in the small intestines through a carrier-mediated mechanism. Illness/infection and high phytate-containing foods are shown to hinder zinc bioavailability by inhibiting uptake [18,19]. The vast majority of zinc found within the human body is stored in skeletal muscle (60%) but reserves are also found in bone (30%), skin and liver (5%) and other organs (2–3%) [13]. Due to the ubiquitous and multifaceted nature of zinc, the effects of zinc deficiency are widespread and impact many organ systems and tissues. Globally, zinc deficiency is a common plight. This may be the combined result of hereditary or dietary problems and can manifest clinically as many disorders including gastro-intestinal (GI) tract malabsorption syndromes [20], liver and renal diseases [21], aging [22], immune dysfunction [22,23], dermatitis [24], mental and growth retardation [25,26], hypogonadism [27] and impaired wound healing [28].

Studies dating back to 1970 and earlier have shown the importance of zinc concentrations towards healing wounds in patients with thermal injuries or exposure to surgical stress [29]. Zinc is especially important in skin [30]. Skin contains a relatively high (about 5% of body content) zinc content, primarily associated within the epidermis (50–70 μg/g dry weight) [31]. Due to its abundance in the epidermis, mild zinc deficiency is noted to lead to roughened skin and impaired wound healing [32]. Severe zinc deficiency was found to be related to Acrodermatitis enteropathica (AE), a potentially fatal genetic disorder in which individuals are unable to absorb sufficient dietary zinc as a result of mutations in the SLC39A4 gene which encodes hZIP4 [33]. Zinc deficiency associated with AE leads to dermatitis, diarrhoea, secondary bacterial/fungal infections and often mortality in untreated infants. AE patients’ symptoms can be dramatically improved via dietary zinc supplements [34].

Zinc deficiency has been linked to delayed wound healing [32,35] and low serum zinc levels have been reported in critically ill patients within Intensive Care Units [36]. Although the benefits of supplemental zinc have been documented in critically ill patients [37], severe burn injury [38,39], subcutaneous abscess, minor surgery [40] and pressure ulcers [41,42], the effects of zinc on wound healing has only been minimally reviewed [32,35].

Wound healing, inflammation and immune response are intimately associated with one another. Over the years zinc has been shown capable of modulating both innate and adaptive immune functions. Zinc alters immune responses in a multitude of ways ranging from myeloid-derived cells and inflammatory signalling to lymphocyte differentiation and antibody production. The ability of zinc to regulate immune homeostasis is a burgeoning field of study and has been extensively reviewed by others [23]. Here, we discuss the contributions of zinc within the wound repair processes with an emphasis on platelet cells and haemostasis, inflammation and host defence, granulation and re-epithelization, ECM remodelling via regulation of matrix metalloproteinases (MMPs) and its association with tripartite motif (TRIM) family proteins in membrane repair and wound healing [43,44]. By identifying roles by which zinc coordinates wound healing, we hope to stimulate further research in tissue repair and regeneration.

## 2. The Phases of Wound Healing and Zinc’s Impact

**Figure 1:**
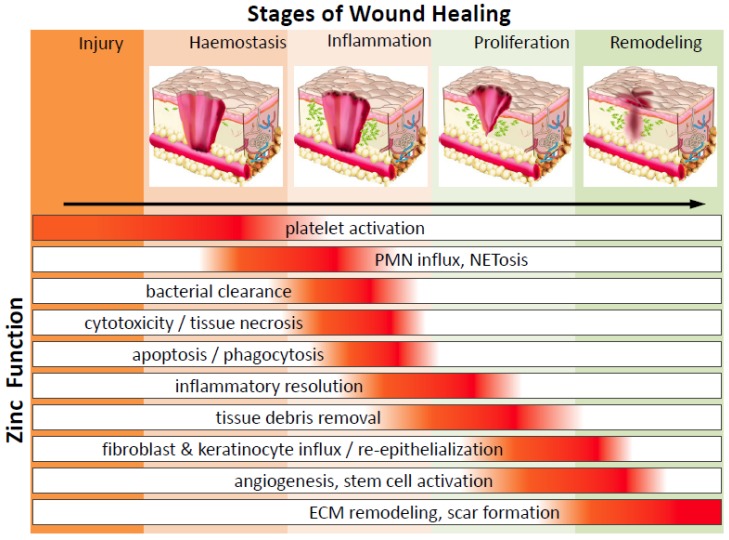
The function of zinc throughout the stages of wound healing. Zinc has a significant function in many cells over the entire process of wound repair. After injury wounded tissue must establish haemostasis via coagulation and clot formation. Injury is quickly followed by immune infiltration and inflammation as a means to clear the wound of damaged tissue and microbes, thus preventing infection and allowing room for granulation. Fibroblast, epithelial cells, keratinocytes and endothelial cells, will proliferate and migrate into wounds to deposit ECM and re-populate the injury site, facilitating wound closure. Finally, matrix deposition and clearance regulates the development of scar formation. PMN—polymorphonuclear leukocytes; NETosis—a novel form of programed neutrophil death that resulted in the formation of neutrophil extracellular traps (NETs); ECM—extracellular matrix.

Wound healing is an intricate and dynamic process which can be subdivided into a series of phases including: (1) coagulating fibrin clot formation (haemostasis, occurs within seconds to 1 h), (2) inflammatory response (within minutes to days), (3) cell proliferation, re-epithelialization, granulation and angiogenesis (begins 18–24 h after wounding and lasts from days to weeks) and (4) matrix remodelling and scar formation (5–7 days after injury and could persist months to years) (Figure 1). As detailed below, these distinct but overlapping phases involve dynamic coordination of soluble mediators (reactive oxygen species (ROS), chemokines, cytokines and growth factors), ECM turnover and cellular cross-talk amongst platelets, infiltrating immune cells, resident keratinocytes, endothelial cells, fibroblasts, epithelial cells and stem cells. Within this section we will closely examine the impact of zinc regulation on wound healing.

### 2.1. Haemostasis and Platelets

Serious injury results in vasculature damage and it becomes imperative to stanch bleeding. The major mechanism by which haemostasis is accomplished is via activation and aggregation of platelet cells. Platelets are capable of responding to vascular damage by means of aggregating at the damage site to form an initial clot followed by fibrin deposition and polymerization to further strengthen the plug [45]. It has been understood for decades now that zinc is capable of enhancing platelet activity and aggregation [46,47]. Recently it was shown that the effect of zinc on platelets is mediated through Protein kinase C (PKC)-mediated tyrosine phosphorylation of platelet proteins [48,49]. Exogenous zinc treatment (at millimolar concentration range) induced zinc entry into the platelet cytosol and time-dependent (within 30 min) stimulation of tyrosine phosphorylation on certain high molecular weight proteins which could be blocked by PKC inhibitors. The intriguing role of zinc on pathophysiological thrombus formation during tissue injury is still largely unknown. Platelets are increasingly being recognized as immune cells capable of pathogen recognition and mediating an inflammatory response via cytokines and chemokines [50]. Platelets contain alpha-granules, which carry a plethora of proteins and factors, such as Chemokine (C-X-C motif) ligand 1 (CXCL1, GRO-α (growth regulated α protein), CXCL4, CXCL5 (ENA-78 ,epithelial-derived neutrophil-activating protein 78), CXCL7 (PPBP (Pro-Platelet basic protein), β-TG (Beta-Thromboglobulin), CTAP-III (connective tissue activating peptide III), NAP-2 (neutrophil-activating peptide-2)), CXCL8 (IL-8, interleukin-8), CXCL12 (SDF-1α, stromal cell-derived factor-1α), Chemokine (C-C motif) ligand 2 (CCL2, MCP-1(monocyte chemoattractant protein-1)), CCL3 (MIP-1α, macrophage inflammatory protein 1-α) and CCL5 (RANTES, regulated upon activation normal T cell expressed and secreted); factors capable of recruiting and activating innate immune cells to the wound site [51]. It has been shown that zinc induces alpha-granule release [48]. These studies suggest that platelets and zinc play an important role in initiating the inflammatory phase of wound healing.

### 2.2. Inflammation and Immune Defence

During and after haemostasis, wounded tissue undergoes an inflammatory stage. This is an elaborate process involving coordination amongst a diversity of cell types. Innate immune cells migrate to and infiltrate injured tissue whereby they can rid the environment of cellular debris and infectious microbes. In experimental human studies of mild zinc deficiency, the affected elderly subjects (ages 55–85 years) had increased inflammatory cytokines and oxidative stress production [52]. Zinc supplementation in elderly subjects reduced plasma levels of oxidative stress markers, decreased ex vivo production of inflammatory cytokines like C-Reactive Protein (CRP) and IL-6, reduced chemokines such as monocyte chemoattractant protein-1 (MCP-1) and reduced secretary cell adhesion molecules (soluble vascular cell adhesion molecule-1 (sVCAM-1), soluble intercellular adhesion molecule-1 (sICAM-1) and s-E-selectin) which represent important biomarkers of cell damage-associated inflammation in endothelium and platelets [18,52,53]. Inflammation-associated vascular dysfunction could be due to peroxisome proliferator-activated receptors (PPAR) dysregulation during zinc deficiency [54].

Neutrophils are granular polymorphonuclear leukocytes (PMN), which often act as one of the first responders to tissue injury and bacterial infection. The migration of neutrophils and other leukocytes to infected/damaged sites is accomplished via increasing gradients of chemokines and cytokines, a process known as chemotaxis. In rhesus monkeys, zinc deficiency obstructs PMN chemotaxis while supplementation reverses the effects [55]. Upon arrival to wounds neutrophils are capable of secreting inflammatory cytokines and phagocytizing pathogens in order to prevent infection. In a study of zinc-deficient acquired immune deficiency syndrome (AIDS) patients, zinc supplementation was observed to enhance neutrophilic phagocytosis of opsonized zymosan particles, a Toll-like receptor-2 (TLR2) agonist [56].

Phagocytes, such as neutrophils, are able to destroy intracellular pathogens via ROS. Enzymes important for the generation of ROS precursors and bacterial clearance, are nicotinamide adenine dinucleotide phosphate (NADPH)-oxidases. Interestingly excess zinc, as well as zinc-deficiency, can both inhibit NADPH-oxidases and thereby hinder microbial elimination [57,58]. A unique mechanism by which neutrophils are able to eliminate pathogens is via the release of neutrophil extracellular traps (NETs). NETs are extracellular matrices consisting of granule proteins, chromatin and DNA; capable of trapping and killing microbes [59]. Absence of zinc, via chelators, compromises NETosis [60]. The contents of NETs include heterodimeric proteins known as calprotectin [61]. Calprotectin is able to bind and sequester zinc ions [62]. By sequestering zinc, calprotectin deprives bacteria of essential metals and hinders bacterial survival and expansion. This method of microbial control falls under a category known as “Nutritional Immunity”, a process in which host cells sequester and limit the availability of essential trace elements thereby inhibiting pathogenic growth. Bacteria have developed their own response to combat strategies such as calprotectin. Some bacteria have evolved to express zinc uptake receptors with higher zinc-affinity than calprotectin, or even receptors that bind and repurpose calprotectin, thus allowing them to evade nutritional immunity [63,64].

Monocytes are pro-inflammatory innate immune cells. Monocytes respond to chemokines and migrate to affected tissue where they adhere to endothelial cells, infiltrate tissue and differentiate into macrophages capable of clearing pathogens/damaged tissue and propagating inflammation. It has been reported that zinc has conflicting roles on monocyte/endothelial adhesion. It has been shown that zinc-deficiency as well as zinc oxide treatment result in elevated monocyte adhesion [65,66]. Dierichs et al. recently reported that zinc could participate in modulation of monocyte differentiation into pro-inflammatory (M1) or immune-regulatory/wound healing (M2) macrophages [67]. M1 macrophages are important for early inflammation and microbial/debris clearance, while M2 macrophages are involved in immune suppression and later tissue remodelling/repair. Using the human monocyte cell line THP-1s (Tohoku Hospital Pediatrics-1), they discovered that both zinc deficiency and supplementation promote M1 phenotypes, while inhibiting M2 differentiation. Counterintuitively, they also report that zinc supplementation suppresses inducible nitric oxide synthases (iNOS) expression, an M1 hallmark associated with reactive nitrogen species production and pathogen clearance. Maintaining an appropriate balance between M1/M2 macrophage populations is complex and crucial during wound healing. Fully elucidating the effect of zinc on macrophage phenotypes and functions will aid in the advancement of wound healing treatments.

Macrophages eliminate inflammatory pathogens by phagocytosis, a process that microbes are taken up and trapped via membrane bound intracellular phagosomes. From there microbes can be killed by means of oxidation or lysosomal degradation. Phagosomes can fuse with lysosomes, whereby the protease-containing acidic environment can destroy bacteria. Many bacteria have developed strategies whereby they can live within intracellular phagosomes undetected and prevent lysosome fusion/degradation. Under these circumstances, similar to neutrophils, macrophages have developed their own forms of nutritional immunity. Through the use of zinc transporters, macrophages are able to shuttle zinc in or out of bacterial laden phagosomes. Depending on the microbes present, macrophages are able to deprive bacteria of zinc, essentially starving them, or alternatively poison bacteria with toxic levels of zinc and other heavy metals [68,69]. *M. tuberculosis* developed a defence to zinc toxicity however, via P-type ATPases, capable of exporting zinc and maintaining viable ion concentrations even within the toxic environment of phagosomes [70].

Macrophages and other immune cells, are major producers of cytokines. Many studies have shown that zinc modulation has an effect on cytokine production. Some of the major regulators of inflammatory cytokine production are T cell receptor (TLR) proteins. TLR proteins are capable of recognizing pathogen-specific molecular motifs and implement signalling cascades. Closely associated with TLRs is nuclear factor kappa-light-chain-enhancer of activated B cells (NF-κB) signalling. NF-κB is a potent transcriptional regulator involved in a plethora of cellular processes associated with wound healing including: inflammatory response, tissue remodelling, proliferation, apoptosis, cell adhesion, etc. [71]. Numerous reports have detailed the effects of zinc on TLR/NF-κB mediated inflammatory signalling, however studies have come to conflicting conclusions. Hasse et al. reported that lipopolysaccharide (LPS)/TLR4 mediated NF-κB signalling is dependent upon intracellular free zinc [72]. In their study sequestration of zinc via an intracellular membrane-permeable zinc ion chelator, TPEN (*N*,*N*,*N*′,*N*′-tetrakis(2-pyridinylmethyl)-1,2-ethanediamine), completely abolishes NF-κB activation after LPS stimulus. In contrast, there is a growing body of evidence that zinc acts as an inhibitor of TLR/NF-κB signalling. Reports have shown that zinc is capable of negatively regulating NF-κB signalling via PPAR-α, A20, IκB kinase-β (IKKβ) and phosphodiesterase (PDE) [73,74,75,76].

Dynamic crosstalk exists between zinc homeostasis and inflammation. A negative feedback loop appears to exist within zinc/NF-κB signalling. It has been demonstrated that upon inflammatory stimulation NF-κB upregulates the zinc transporter ZIP8. ZIP8 translocates to the plasma membrane where it facilitates zinc uptake into the cell. Intracellular zinc is thereupon free to inhibit IKKβ and negatively regulate the inflammatory process [75]. In a similar negative feedback loop, IL-6 stimulus results in upregulation of ZIP14, which is also capable of attenuating inflammation in hepatocytes [77].

IL-1β and IL-18 are potent pro-inflammatory cytokines under the regulation of caspase 1 activation. Caspase-1 belongs to the pro-apoptotic endoprotease family of Caspases. Research has yielded conflicting results on the pro vs. anti-effects of zinc on apoptosis. While results have been ambiguous, zinc concentration appears to be an important factor [78]. Groups have shown that zinc has a direct inhibitory effect on the activity of caspases 3, 6, 7, 8 and 9 [79,80,81]. A similar study utilizing a zinc-containing compound, ziram, showed pro-caspase1, the inactive precursor of caspase1, degraded upon ziram treatment [82]. Degradation of the pro-inflammatory precursor (pro-caspase1) indicates a potential role for zinc in regulating caspase-mediated inflammation. This notion is supported by clinical studies showing higher levels of IL-1β expression in overweight patients with low dietary zinc intake, compared to patients with higher zinc intake [83].

As mentioned earlier, macrophages partake in the clearance of not only microbes but also damaged tissue. Lymphocytes and the adaptive immune system also play an important role in this element of wound healing. It has been shown that B-lymphocytes aid in wound clearance and repair [84,85]. Mature B-cells/plasma cells are capable of producing antibodies that detect injured tissue. These antibodies serve as signals by which macrophages recognize and phagocytize damaged cells. Zinc deficiency results in lowered populations of both precursor and mature B-cells and can reduce antibody production [86]. Diminishing B-cells populations and ergo circulating antibodies, would negatively affect phagocytosis causing hindered wound clearance and chronic wounds. In fact, it has been demonstrated in chronic diabetic skin lesions, that direct B-cell treatment accelerates wound healing [87].

### 2.3. Inflammatory Resolution and Tissue Growth (Proliferation) Stage

During wound healing, it is important to resolve inflammation and initiate re-epithelization, the process where epithelial cells proliferate and repopulate injured tissue for wound closure. M2 macrophages are one cell type that helps mitigate inflammation but there are numerous other immune cells that aid in this process. Zinc deficiency also has a significant impact on T lymphocyte populations [86,88]. Regulatory T lymphocytes (Tregs) regulate and suppress inflammation. Zinc supplementation is known to increase the number of Tregs in multiple circumstances: mixed lymphocyte cultures, in response to allergens and when combined with transforming growth factor-β (TGFβ) [89,90,91]. Cutaneous wound studies have shown that Tregs help resolve inflammation, promote re-epithelization and wound contraction [92]. It would be interesting to determine whether zinc supplementation is capable of upregulating a regulatory T-cell response in order to promote accelerated wound repair.

About two or three days after the wound occurs, fibroblasts begin to enter the wound site, marking the onset of the tissue proliferative phase. Fibroblast infiltration is associated with collagen/ECM deposition, which serves as a temporary scaffold for repair. Collagen serves as a bed enabling migration of epithelium, keratinocytes and microvasculature. One of the primary regulators of ECM deposition and fibrosis is TGFβ/SMAD (Mothers against decapentaplegic homolog protein) signalling. Zinc is a vital cofactor for SMAD signalling and thus plays a major role in formation of granulation tissue [91]. During re-epithelialization and wound closure, there is transient proliferation and migration of resident keratinocytes, fibroblasts, epithelial cells and endothelial cells with simultaneous activation and trans-differentiation of multiple epidermal stem cells populations [93]. The collaborative efforts of collagenases and plasminogen activators led to degradation of fibrin clots along with the zinc-dependent matrix metalloproteinase (MMP, discussed in more details below), which digest dermal basal membranes and ECM, allows room for cell growth, migration and angiogenesis.

Within the epidermis, apoptotic and necrotic cells stimulate epidermal cells proliferation and migration over granulation tissue in order to repair the epidermis. Topical zinc application enhanced re-epithelialization in porcine skin wound healing model [94]. Moreover, a study testing galvanic Cu/zinc (Zn) microparticles on wound closure reports ROS-mediated enhancement of human dermal fibroblast migration [95]. Similarly, zinc was shown to increase keratinocyte migration and participate in re-epithelialization of the epidermis [96]. Concurrent with re-epithelialization, endothelial cells migrate and proliferate into wound sites to establish new blood vessels in a process called neovascularization, or angiogenesis, thus supplying essential oxygen and nutrients for the growth of cells in the wound bed. Zinc has been shown effective for angiogenesis in vivo [97]. However, studies analysing the effect of micronutrient trace elements on vascular biology report contradictory findings in which zinc acts as an anti-angiogenic agent altering important genes and growth factors [98]. Further research and characterization is needed regarding zinc and this phase of tissue healing and regeneration.

### 2.4. Wound Resolution and Matrix Remodelling

ECM is composed of a complex mixture of insoluble molecules such as collagens, laminins, fibronectin, integrin and heparin sulphate proteoglycans and provide a solid supportive matrix scaffold for cells. The ECM network also serves as a reservoir of a plethora of cytokines, growth factors and molecular cues, both active and latent, to promote cellular migration, epithelialization, adhesion and wound contraction [99]. Among the many proteins essential for epidermal wound repair are the ECM-remodelling matrix metalloproteinase (MMPs) family proteins. MMPs are secreted by distinct cell types such as inflammatory cells, keratinocytes, endothelial cells and fibroblasts. MMPs are zinc-dependent endopeptidases which act to modulate growth factor activation, cleavage, degradation and composition of ECM, processing of cell-cell junctional adhesion molecules, cytokines and cell surface receptors and cell-matrix signalling during different stages of wound healing [100,101,102,103].

MMPs are synthesized as inactive zymogens (proMMPs) and their functions are modulated in four ways—namely gene expression, compartmentalization, proMMPs activation and MMPs enzymatic inactivation. The structures of MMPs are composed of four domains, the pro-peptide domain with a conserved cysteine switch motif PRCGXPD, the zinc-binding catalytic domain, a hinge region and the *C*-terminal hemopexin-like domain. Activation of MMPs is dependent on a “cysteine switch” mechanism [104]. The pro-peptide cysteine interacts with the catalytically important zinc ion in the active site to keep proMMPs in an enzymatic inactive state to prevent their interaction and cleavage of substrates. The *C*-terminal hemopexin-like domains determine substrate specificity and their interaction with MMP inhibitors, the tissue inhibitor of metalloproteinases (TIMPs). Many stimuli such as tissue injury [105], oxidative stress upon UV exposure [106], inflammatory cytokines such as TNFα or IL-1α [107] and certain growth factors like vascular endothelial growth factor (VEGF) trigger the activation of MMPs [108]. Secreted or cell surface localized TIMPs also play important roles in wound repair. TIMPs process a wide range of extracellular substrates such as ECM proteins, cytokines and their receptors through their regulations of metalloproteinase activities [109]. These intricate activation mechanisms keep MMPs activities under tight control and in dynamic homeostasis under different wound healing demands [110].

Coordinated MMP function is pivotal for ECM degradation, migration of keratinocytes over ECM (wound re-epithelialization) [111], neo-angiogenesis [112], cell proliferation, fibroblast activation/migration and collagen deposition and fibrous tissue (fibroplasia and scar) formation during tissue remodelling to restore epidermal tissue integrity [113]. Prolonged ECM deposition leads to tissue fibrosis, whereas MMPs dysfunction is linked to defective wound closure [100,114]. Zinc and calcium ions are required for optimal MMPs function in vitro [115], however, the molecular mechanism by how zinc regulates MMPs function in vivo is still not fully understood due to the complexity of their regulation and the numerous MMPs involved in wound healing.

## 3. Zinc Is an Antioxidant Micronutrient

Several studies have shown that zinc deficiency increases oxidative stress [22,116,117]. It is well accepted that oxidative damage is a major cause of tissue injury and redox regulation plays a prominent role in wound repair [118,119,120]. Superoxide radicals such as reactive oxygen species (ROS) and reactive nitrogen free radical species (RNS) are by-products of electron leakage during mitochondrial electron transfer and constitute various forms of oxidative stress [121,122]. Superoxide radicals can cause oxidative damage to biomolecules, such as DNA, protein and lipids, thus impairing their bioactivity.

In biology, zinc is redox-inert and opposes the absorption and activity of redox-active transition metals like copper and iron, which catalyse the production of highly reactive oxidative stress [123,124]. Metallothionein (MT) is a family of low molecular weight, cysteine-rich (cysteine constitutes 30% of MT amino acids) proteins capable of binding heavy metals through their sulfhydryl (-SH)-rich cysteine residues. A large percentage (nearly 20%) of intracellular zinc is bound to MTs and can be rapidly released according to physiological needs. MTs are redox-sensitive antioxidant proteins, crucial for zinc regulation and protection against heavy-metal toxicity and oxidative stress. Interestingly, synthesis of MTs is dependent on availability of the dietary zinc, copper and selenium [125]. Zinc exhibits acute antioxidant properties by means of binding to and thus protecting redox-sensitive sulfhydryl groups of MTs [124,125]. Zinc supplementation induces MT expression and has been shown to protect against UV immunosuppression [126]. Oxidative stress associated with long term zinc deficiencies can be attributed to a reduction in the synthesis of MTs and a decline in the antioxidant activity of zinc-containing Cu, Zn superoxide dismutase (SOD1) [127].

## 4. TRIM Proteins in Membrane Repair and Wound Healing

Tripartite motif family (TRIM) proteins are characterized by the presence of *N*-terminal Ring (Really Interesting New Gene)-finger domain, zinc-finger B-box domains and a coil-coil domain. There are over 80 identified human TRIM proteins which play important roles in regulating cellular processes such as protein degradation, innate immunity, cell survival/death, oncogenesis, development and intracellular signalling. TRIM proteins exhibit a wide breadth of diversity along their *C*-termini, resulting in 12 different subfamilies/classifications. Most TRIM proteins contain E3 ubiquitin ligase activity, a class of enzymes which catalyze the final step (E3 step) in the ubiquitination cascade to form an ubiquitin covalent bond with a substrate lysine. TRIM proteins have been widely studied and reviewed within the context of immunology, cell death/survival and cancer [128,129,130,131], however an emerging role in wound healing has developed.

Maintaining cell membrane integrity is vital to normal cell physiology and function [132,133,134]. Repair of damaged cellular membranes requires intracellular vesicle trafficking which leads to an accumulation of vesicles close to the plasma membrane [135]. Subsequent resealing of the cell membrane is imperative for the survival and long-term viability of cells. Disruption of membrane repair contributes to pathophysiological conditions such as dystrophic muscle [136], diabetes [137], poor wound healing, chronic ulcer and scarring [133,134].

We have previously identified a novel TRIM protein, Mitsugumin 53 (MG53) or TRIM72, as an essential component of the cell membrane repair machinery [138,140] and is highly expressed in muscle. MG53 is capable of sensing oxidative stress associated with cell membrane damage and responds by binding to phosphatidylserine containing intracellular vesicles and then shuttles pre-assembled sub-membrane/intracellular vesicles to patch plasma membrane disruptions caused by mechanical or chemical injury [138,139,140]. Furthermore, MG53 is capable of protecting against ROS-mediated cellular stress, as seen with ischemic reperfusion injuries and metabolic disorders and prevents cell death [141]. MG53 ablation in mice (*MG53*^−/−^) results in defective membrane repair with progressive pathological consequences in skeletal and cardiac muscle and enhanced susceptibility to injuries in lungs, kidneys and skin [43,142,143,144,145,146,147,148]. The protective repair function of endogenous and recombinant human MG53 (rhMG53) protein have been reported in multiple tissue types ranging from skeletal muscle [136,144,149], heart [141,145,146,150], kidney [142], lung [143,151], skin [43,44] and brain [147].

The binding of zinc to TRIM family proteins is critical to their E3 ligase activities [152]. While membrane repair mechanisms can be context dependent, we have shown that zinc serves as a molecular switch linking oxidative stress and the membrane reseal function of MG53 [153]. MG53 consists of two zinc binding domains in the Ring-finger and B-box motifs [138,153]. Zinc binding appears to be essential for MG53-mediated membrane repair, as mutation of either zinc binding residue compromises the membrane resealing ability of MG53. Simultaneous disruption of both zinc binding motifs in double mutants further exacerbates this effect [153]. Moreover, defective MG53-mediated vesicular transport and membrane repair was observed in the absence of extracellular zinc via a biochemical zinc chelator. These results suggest that MG53 acts as an acceptor for zinc during cell membrane repair.

**Figure 2:**
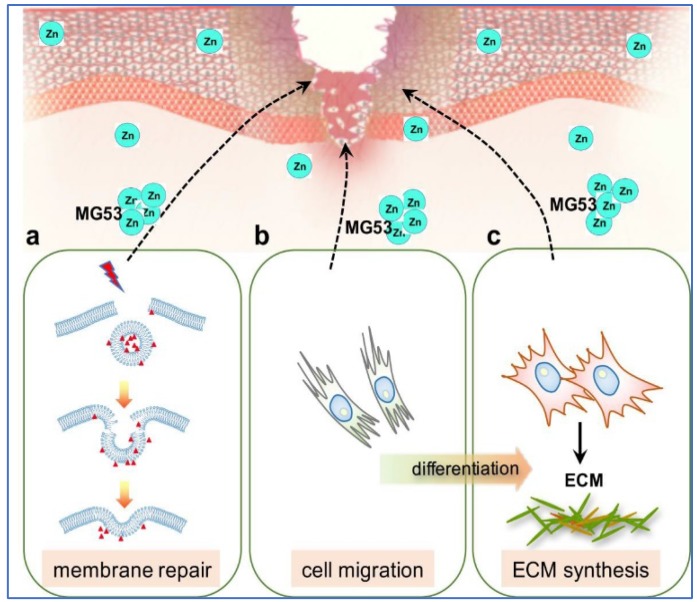
Proposed roles of zinc in MG53 mediated wound healing process. MG53 exists as intrinsic intracellular vesicles which could be secreted into blood circulation as a myokine to mediate wound healing. In response to dermal injury, MG53 could exert the following functions. (**a**) MG53-containing vesicles nucleate the cell membrane repair machinery and trafficking to damaged cell membrane to protect against acute membrane injury of keratinocytes and fibroblasts; (**b**) MG53 mediates cytoskeletal stress fibre remodelling to promote fibroblasts migration to the wound sites; and (**c**) MG53 regulates TGFβ signalling to modulate trans-differentiation of fibroblasts into myofibroblasts and suppresses deposition of ECM proteins during tissue remodelling stage of wound healing. Zinc is proposed to bind to MG53 on its two zinc-binding sites and modulates MG53-mediated wound healing process (Modified from JBC 290(40): 24592). MG53: Mitsugumin 53; ECM: extracellular matrix; TGFβ: transforming growth factor-β.

Current reports support the findings that TGFβ plays a central role throughout wound healing [154,155,156]. Excessive TGFβ signalling can lead to maladaptive repair conditions and tissue fibrosis [157]. Recently, we showed MG53 is a vital component of wound healing and topical application of rhMG53 protein promotes wound healing with reduced scar formation [43,44]. MG53 deficiency, in mice, leads to delayed wound healing with aberrant scar formation, while therapeutic treatment with rhMG53 accelerates healing and prevents scarring. In vitro assays revealed that rhMG53 induces fibroblast migration while simultaneously suppressing myofibroblast differentiation and production of fibrotic proteins. The underlying molecular mechanisms of MG53-mediated protection against dermal wounding involves membrane repair, modulation of cell migration and suppression of ECM protein synthesis via inhibition of TGFβ/SMAD signalling (Figure 2).

MG53 is a versatile molecule with function associated with various signalling pathways like reperfusion injury salvage kinase (RISK) [145], glycogen synthase kinase-3β (GSK3β) [136,145,158], cell survival kinase AKT, TGFβ [43] and modulation of inflammatory mediators [143]. These findings are promising with regards to MG53s translational potential in wound healing and tissue regeneration. However, how zinc impacts MG53 E3-ubiquitin ligase activity and the identities of the E3-ligase substrates involved in MG53-mediated wound healing remain elusive and requires further investigation.

Two other TRIM proteins have been identified that might play a role in wound healing. Beer et al. reported that TRIM16, also known as oestrogen-responsive B box protein (EBBP), is a regulator of keratinocyte differentiation. TRIM16 expression is downregulated in skin wounds and hyper-thickened epithelium [159]. Over expression of TRIM16 enhances early differentiation of keratinocytes and is believed to regulate the differentiation capacity of cells. TRIM16 has extensively been studied in the field of cancer but little attention as has been directed toward wound healing. Interestingly, a study on ovarian cancer metastasis showed that overexpression of TRIM16 inhibited expression of MMP2 and MMP9 [160]. It is possible that TRIM16 is downregulated during wound healing in order to allow for tissue granulation, re-epithelialization and remodelling.

Recently the function of TRIM28 in endothelium was investigated by Wang et al. They show that TRIM28 helps mediate not only inflammation but also angiogenesis in a VEGFR2 dependent manner. Scratch wound assay showed endothelial cell migration was significantly reduced, after TRIM28 siRNA treatment [161]. These findings have significant implications for inflammation and wound healing. While both TRIM16 and TRIM28 have promising potential for wound repair, they are yet to be directly applied to such a study. Additionally, characterizing the impact of zinc on these proteins’ functions would be beneficial. Further research into zinc regulation of TRIM protein expression/function during wound healing would provide new and exciting insight on wound repair.

## 5. Zinc in Wound Healing—Clinical Perspectives

The role zinc plays in wound healing can be viewed from two perspectives: first, the impact of zinc deficiency and second, the effect of zinc supplementation (topical/local or systemic) on wound repair. The association between zinc deficiency and delayed wound healing has been described [32,35]. Treating zinc deficiency results in improved wound healing compared to those with zinc deficiency. For example, in patients considered to be at risk of refeeding syndrome, it may be appropriate to give a loading dose of 10–30 mg of Zn, followed by the daily maintenance dose of 2.5–5 mg [4]. However, the impact of zinc supplementation on wound healing in patients without zinc deficiency is less well known. There are very few well-done clinical studies on the topic and what little information currently available is inconsistent. For example, the Cochrane database reports 6 small studies on patients with arterial or venous ulcers and found that oral zinc supplementation did not improve wound healing [162]. Contrarily, another meta-analysis of topical zinc therapy with zinc oxide paste-medicated dressing containing zinc concentration between 6–15% for chronic venous leg ulcers showed improved healing, though the authors point out that the studies were small and of sub-optimal quality [163]. Cereda and co-authors performed a randomized, prospective trial in malnourished patients with chronic pressure ulcers [164]. They found a significant reduction in the size of the ulcers after 12 weeks of supplementation with a high calorie, high protein oral formula that was also supplemented with arginine, anti-oxidants and zinc (either orally or tube fed with 18–20 mg zinc daily). It is unknown how significant of a role the zinc supplementation played in this study. Attia and colleagues reported on 90 non-diabetic patients with uncomplicated wounds who were topically treated with one of two zinc-containing fluids (regular crystalline insulin or aqueous zinc chloride solutions at 0.2 mg/100 mL per 10 cm^2^ wound) versus a control of 0.9% normal saline. The groups treated with the zinc-containing fluids had significantly improved healing [165]. On the other hand, a study of 42 patients with pressure ulcers treated with oral l-carnosine versus zinc containing polaprezinc (at 34 mg per day) showed no difference in healing [166]. It is clear that more studies utilizing more stringent controls will be necessary to fully understand the clinical potential of zinc supplementation.

Due to the loss of zinc during injury, zinc therapy has been used in wound care to enhance healing in zinc-deficient patients [32,167]. Topical zinc sulphate (ZnSO_4_) application, usually at an optimal 3% concentration, has been widely used in wound healing for its antioxidant effect [30]. Other forms of application include 1% ZnCl_2_ or the largely insoluble zinc oxide (ZnO). ZnO provides prolonged supply of zinc to wounds and enhance its healing ability. Additionally, ZnO increases collagen degradation in necrotic wounds [168]. It has been shown that topical zinc application induces mRNA expression of metallothionein, which could account for its anti-UV photoprotective effect [30,169]. A standard regimen for severe burn care includes regular daily dietary zinc supplementation equivalent or exceeding 22 mg [39,170]. Moreover, recent advances in drug delivery with zinc oxide nanoparticle (ZnO-NPs) technology has received considerable attention for the treatment of wounds due to their effective cell penetration, immunomodulation and antimicrobial capacity [171,172]. However, in-depth pharmacodynamics and toxicology studies are still needed prior to widespread applications [173].

## 6. Concluding Remarks and Future Perspectives

Zinc is a micronutrient that is essential to human health. Zinc plays a major role in regulating every phase of the wound healing process; ranging from membrane repair, oxidative stress, coagulation, inflammation and immune defence, tissue re-epithelialization, angiogenesis, to fibrosis/scar formation. With huge demands for improved wound care, we need a more thorough in-depth understanding of the molecular mechanisms in which zinc functions. A more thorough comprehension of the cellular cross-talks and MMP regulation would go a long way in progressing the field wound healing. The biological function and therapeutic potential of TRIM proteins, such as MG53, in wound repair is emerging. Understanding the precise mechanisms by which zinc regulates their activity remains unexplored, yet is crucial. Further inquiry into the mechanisms of zinc and the proteins for which it serves as a cofactor, will greatly advance the treatment and care of difficult-to-heal wounds.

## Abbreviations

AE	Acrodermatitis enteropathica
CAM	Cell adhesion molecules
CXCL	Chemokine (C-X-C motif) ligand
CCL	Chemokine (C-C motif) ligand
CRP	C-reactive protein
ECM	Extracellular matrix
ILs	Interleukins
MCP-1	Monocyte chemoattractant protein-1
MMP	Matrix metalloproteinase
MG53	Mitsugumin 53
MT	Metallothioneins
NF-κB	Nuclear factor kappa-light-chain-enhancer of activated B cells
PPARα	Peroxisome proliferator activated receptors-a
PMN	Polymorphonuclear leukocytes
NET	Neutrophil extracellular traps
NADPH	Nicotinamide adenine dinucleotide phosphate
ROS	Reactive oxygen species
TIMP	Tissue inhibitor of metalloproteinases
TLR	Toll-like receptor
TRIM	Tripartite motif
TGFβ	Transforming Growth Factor-β
VEGF	Vascular endothelial growth factor
ZIPs	Zinc transporter proteins
